# Supplementary material for: A Global Screen for Assembly State Changes of the Mitotic Proteome by SEC-SWATH-MS
Source: Cell Syst. 2020 Feb 26;10(2):133–155.e6. doi: 10.1016/j.cels.2020.01.001 (PMC7042714; doi:10.1016/j.cels.2020.01.001)

P43034 | LIS1_HUMAN | PAFAH1B1 LIS1 MDCR MDS PAFAHA
Monomer MW [kDa]: 46.638 Monomer expected elution fraction: 47

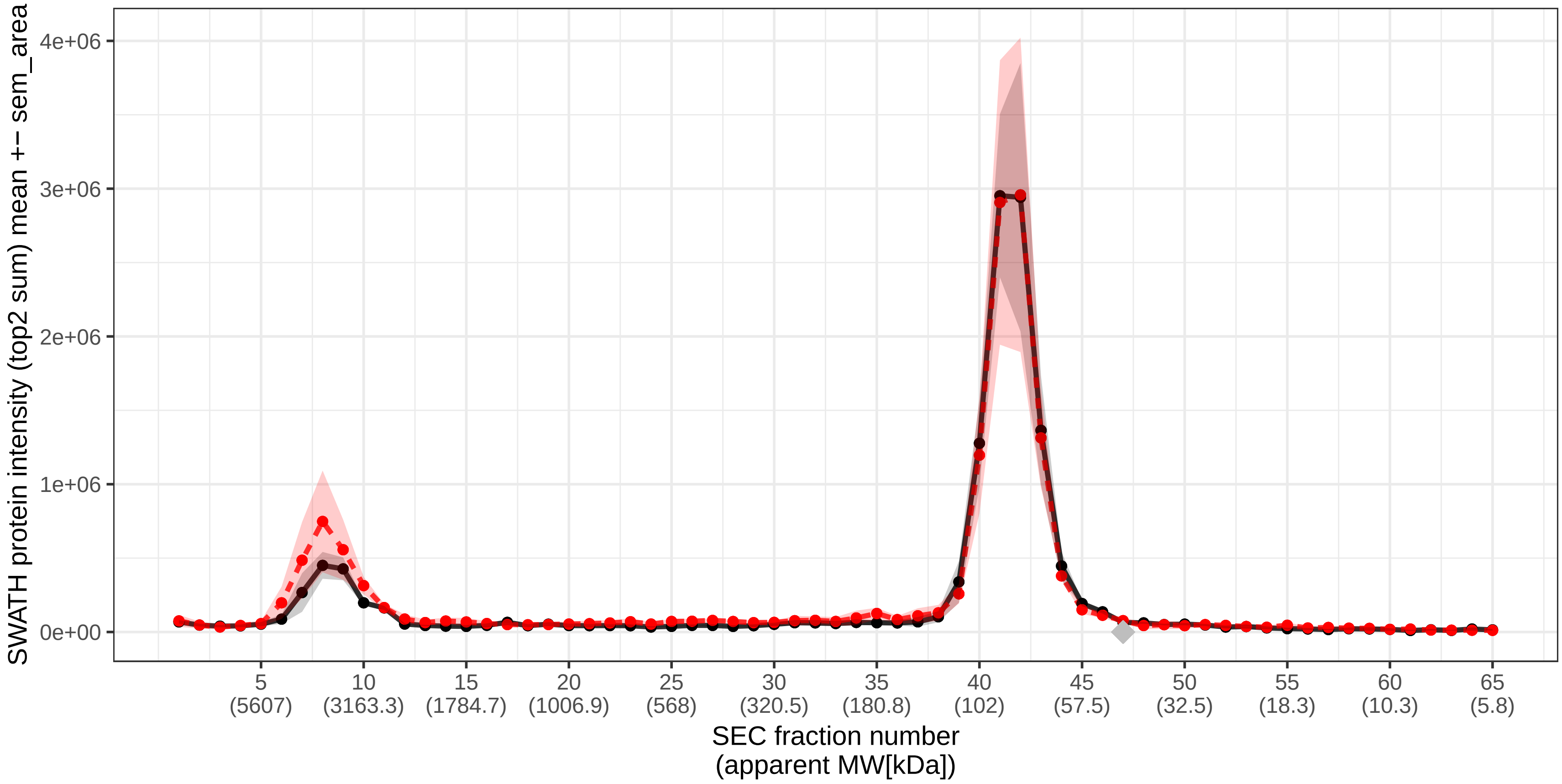

Supplement: Data S1. SEC-SWATH-MS Protein Chromatograms, Related to Figure 1 [file mmc6.zip › SECchrom_P43034_LIS1_HUMAN_PAFAH1B1_LIS1_MDCR_MDS_PAFAHA.pdf]
